# The Unhappy Effects of the Antidepressant Fluoxetine on the Freshwater Microalga *Raphidocelis subcapitata*

**DOI:** 10.3390/toxics13100876

**Published:** 2025-10-14

**Authors:** Manuela D. Machado, Eduardo V. Soares

**Affiliations:** 1 CIETI—Bioengineering Laboratory, ISEP, Polytechnic of Porto, Rua Dr. António Bernardino de Almeida, 4249-015 Porto, Portugal; mmmachado@net.sapo.pt; 2 CEB—Centre of Biological Engineering, University of Minho, Campus de Gualtar, 4710-057 Braga, Portugal; 3 LABBELS—Associate Laboratory, Braga/Guimarães, Portugal

**Keywords:** intracellular ROS accumulation, lipid peroxidation, mitochondrial dysfunction, oxidative stress biomarkers, photosynthetic activity, *Pseudokirchneriella subcapitata* (=*Selenastrum capricornutum*), toxic mode of action

## Abstract

Pharmaceuticals can be found in the aquatic environment and cause unwanted effects on organisms. The present work aimed to characterize the toxic mode of action of the antidepressant fluoxetine (FLX) on the freshwater microalga *Raphidocelis subcapitata*. With this aim, the microalga was exposed to low levels (µg/L) of FLX for 72 h. Exposure to 20–30 µg/L FLX arrested algal growth, which can be explained by the blockage of algal nuclear division. In addition, FLX (15–30 µg/L) deeply altered the alga’s metabolism, which was reflected by an increase in esterase activity, mitochondrial dysfunction (hyperpolarization of inner mitochondrial membrane), and reduction in the content of photosynthetic pigments: chlorophyll *a* (chl*a*) and carotenoids (car). A sharp decline in photosynthetic performance, revealed by the reduction in maximum photochemical quantum yield (*F*_v_*/F*_m_), effective photochemical quantum yield (ΦPSII), and photosynthetic electron transport rate (*ETR*) of photosystem II (PSII), was also observed. FLX, at 30 µg/L, induced the intracellular accumulation of reactive oxygen species (ROS) and lipid peroxidation, with a marginal loss (1%) of cell membrane integrity. The results presented here contribute to the elucidation of the toxic mode of action of FLX on the microalgae *R. subcapitata* and, simultaneously, warn of the negative impact of the presence of pharmaceutical compounds in freshwater aquatic environments.

## 1. Introduction

Pharmaceuticals are biologically active (and often persistent) molecules that, when released into the environment, can negatively impact organisms they were not intended to affect. These substances can enter the aquatic environment either through direct human excretion, when they are eliminated unchanged, or through improper disposal practice, such as flushing expired or unwanted medications down the toilet [1], among which fluoxetine (FLX), an antidepressant belonging to the selective serotonin reuptake inhibitor class, should be mentioned [2]. Since its approval by the U.S. Food and Drug Administration, in the 1980s, this compound has been widely used in treating a wide spectrum of psychological conditions [3]. In 2022, more than 24 million prescriptions of FLX were made in the USA, which corresponded to the 22nd most prescribed medication [4].

Given that FLX is widely used and taking into account its stability in aqueous solution (hydrolytically and photolytically stable) and biological recalcitrance [5], its presence and persistence in municipal effluents, wastewater treatments plants, and groundwater and surface waters, in different parts of the world, is not surprising. For example, FLX has been detected in aquatic environments in Canada, Hong Kong, Norway, South Korea, and the USA [6,7], particularly near urban areas, at concentration levels ranging from ng/L to μg/L [8]. In aquatic environments, the half-life of FLX ranges from 46.6 to 183.2 days, depending on physicochemical conditions such as pH [9].

The observation that birds presented altered behaviour and physiology, after exposure to environmentally relevant concentrations of FLX, highlights the potential negative impact of this antidepressant to bioaccumulate through the food chain [10]. In fact, FLX represents a growing concern for environmental biology [11], negatively impacting aquatic organisms, with risk quotients that can reach values around 105 [7]. Examples of aquatic organisms affected by the presence of FLX are crustaceans, like *Ceriodaphnia dubia* [12,13], *Daphnia magna* [12,13,14,15], *Gammarus pulex* [16], and *Hyalella azteca* [14], and different fishes, such as *Oryzias latipes* [12] and *Pimephales promelas* [12,13,17]. In more recent years, the impact of FLX on larvae of the fish *Danio rerio* has been documented [18].

When comparing the susceptibility of aquatic organisms belonging to different trophic levels (algae, crustaceans, and fish) to FLX, a greater susceptibility of algae (~10–20 times) was observed [12,13]. Moreover, microalgae are at the base of the food web, constituting an important instrument in the assessment of the risk of pollutants. Thus, a toxic effect of FLX on different marine and freshwater microalga was described, such as on *Chlorella vulgaris* [19], *Dunaliella tertiolecta* [20], *Raphidocelis subcapitata* [12,13,15,19], *Scenedesmus acutus* [19], *Scenedesmus quadricauda* [19], and *Scenedesmus vacuolatus* [21]. However, these studies evaluated the toxic effect of FLX exclusively through quantification of growth inhibition. Only more recently have some works been dedicated to characterizing the impacts of FLX on microalgae. These studies revealed that FLX causes oxidative stress and photosynthesis inhibition in the green alga *Chlorella pyrenoidosa* [22] and in the diatom *Phaeodactylum tricornutum* [23].

Since the toxic mode of action (MoA) of FLX on non-target organisms, like algal cells, is clearly poorly understood, it is of paramount importance to explore the toxicity mechanisms of FLX on microalgae. This is particularly true for the relevant model alga *R. subcapitata*. This alga has a ubiquitous distribution and can be found all over the world. It is very sensitive to different pollutants, being recommended by several international organizations for the toxicity assessment of chemicals and the evaluation of water quality [24]. In this context, this study evaluates, for the first time, the impact of FLX on different targets of the freshwater microalga *R. subcapitata*, namely cell-cycle progression, membrane integrity, metabolic activity, photosynthetic pigments content, photosynthetic performance, mitochondrial function, and oxidative stress biomarkers (intracellular accumulation of reactive oxygen species, ROS, and lipid peroxidation). Taking into account the results presented, the toxic MoA of FLX on *R. subcapitata* is proposed.

## 2. Materials and Methods

### 2.1. Strain and Culture Conditions

In the present study, it was used the freshwater microalga *Raphidocelis subcapitata* (Korshikov) Nygaard, Komárek, Kristiansen & Skulberg 1987. This microalga is also known as *Pseudokirchneriella subcapitata* and *Selenastrum capricornutum* [25], and was obtained from the Culture Collection of Algae and Protozoa (CCAP, UK), strain 278/4. The alga was maintained in OECD medium [26] containing 20 g/L agar, at 4 °C, in the dark.

Pre-cultures and cultures were prepared as previously described [27]. Briefly, pre-cultures were performed by resuspending 4–5 colonies of *R. subcapitata* in 40 mL of OECD medium, in 100 mL Erlenmeyer flasks. The algae were incubated for 48 h, at 25 °C, with orbital shaking (100 rpm) (CERTOMAT^®^ H, B. Braun, Melsungen, Germany), and under uninterrupted light (using “cool white” fluorescent lamps, colour temperature of 4300 K, OSRAM, Augsburg, Germany), with an intensity of 4000 lux at the surface of the flask. Cultures were obtained by inoculating 400 mL of OECD medium, pH 8.1, in 1 L Erlenmeyer flasks with 5 × 10^4^ cells/mL of pre-cultures; the cells were incubated in the same conditions described for the pre-cultures. After 48 h of incubation, algae, in the exponential phase of growth, were harvested by centrifugation (2500× *g*, 5 min) and concentrated in fresh algal medium for subsequent use.

### 2.2. Exposure of R. subcapitata to FLX

Algae, in the exponential phase of growth (obtained as described in the previous section), at an initial concentration of 1 × 10^5^ cells/mL, were exposed to 15, 20, or 30 µg/L of fluoxetine hydrochloride (CAS number: 56296-78-7; Sigma-Aldrich, St. Louis, MO, USA), in 1 L Erlenmeyer flasks containing OECD medium (total assay volume: 400 mL), at pH 8.1, for 72 h, under the same conditions described for pre-cultures and cultures (Section 2.1). As control, algae were incubated in the same conditions described above but without FLX. A stock solution of 20 mg/L FLX (Sigma-Aldrich) was prepared in deionized water and stored in the dark at 4 °C.

Determination of algal growth kinetics and cell-cycle progression analysis was carried out from three independent experiments, with samples withdrawn at 24, 48, and 72 h. Algae quantification was performed, in quintuplicate, using a cell counter (TC10, Bio-Rad, Singapore). For all other parameters, samples were collected at 72 h. Thus, cells were harvested by centrifugation (2500× *g*, 5 min) and resuspended in OECD medium to determine the nuclei number, cell membrane integrity, metabolic activity, photosynthetic pigments content, photosynthetic performance, and mitochondrial membrane potential or in PBS buffer (100 mM, pH 7.0) to evaluate oxidative stress.

### 2.3. Nuclei Counting

Cell-cycle progression in *R. subcapitata* was assessed by quantifying the number of nuclei as described by Machado and Soares [28]. Algal cells were stained with SYBR Green I (SGI) (Sigma-Aldrich). The probe, initially provided as a 10,000× concentrated solution, was diluted 1:10 in dimethyl sulfoxide (DMSO, Sigma-Aldrich). Algal cells (5 × 10^6^ cells/mL) were incubated with 2.5 × concentrated SGI for 1 h, in the dark, at 25 °C and then observed by an epifluorescence microscope (EM) equipped with a light-emitting diode (LED) illumination system (pE-400, CoolLED, Andover, UK) and a GFP filter set, both from Leica. For each FLX concentration and control (cells not exposed to FLX), a minimum of 400 cells were evaluated in each condition and independent experiment.

### 2.4. Assessment of Cell Membrane Integrity

Cell membrane integrity was assessed using the fluorescent dye SYTOX Green (SG). SG selectively enters cells with compromised plasma membranes, while it is excluded from those with intact membranes. After a 72 h exposure to FLX, algal cells, 1 × 10^6^ cells/mL, were incubated with 0.5 µmol/L SG (Molecular Probes, Eugene, OR, USA) for 20 min, at 25 °C, in the dark [27]. Cells were observed using an EM equipped with an LED illumination system and a GFP set from Leica. For each FLX concentration, a minimum of 400 cells were counted in each independent experiment.

### 2.5. Determination of Metabolic Activity

Metabolic activity was determined by the evaluation of esterase activity using fluorescein diacetate (FDA), as reported by Machado and Soares [29]. Algal cells (5 × 10^5^ cells/mL) were incubated with 20 µmol/L FDA, in the dark, for 40 min, at 25 °C. Controls included cells not exposed to FLX (positive control) and heat-treated cells at 65 °C, for 1 h (negative control). To verify the influence of FLX on FDA hydrolysis, an abiotic assay was also performed. For this purpose, FDA was combined with the higher concentration of FLX tested (30 µg/L), in the absence of cells.

For each condition, fluorescence intensity was then recorded, in five replicates, as relative fluorescence units (RFUs), using a microplate reader (Victor3, Perkin-Elmer, Turku, Finland) with an excitation wavelength of 485/14 nm and an emission wavelength of 535/25 nm.

### 2.6. Evaluation of Pigments Concentration and Photosynthetic Performance

For photosynthetic pigments quantification, 5 mL of algal suspension (3 × 10^6^ cells/mL) was centrifuged 2500× *g* for 10 min. After discarding the supernatant, cells were resuspended in 5 mL of 90% (*v*/*v*) acetone (VWR Chemicals, Fontenay-sous-Bois, France) and pigment extraction was performed at 4 °C, for 20 h. After extraction, the cells were collected by centrifugation at 2500× *g*, for 10 min, and the absorbance of the supernatant was measured at 630, 647, 664, and 750 nm, for chlorophyll *a* (chl*a*) determination [30], and at 480 nm for carotenoids (car) quantification [31]. Pigment quantification was performed in triplicate for all FLX treatments in each independent experiment.

Photosynthetic activity of photosystem II (PSII) was measured using a pulse-amplitude-modulated (PAM) chlorophyll fluorometer (Junior PAM, Walz, Effeltrich, Germany). A dark adaptation period of 30 min was applied to algal cells (3 × 10^6^ cells/mL) before measurement. Subsequently, the minimum fluorescence (*F*_0_) and maximum fluorescence (*F*ₘ) yields were recorded, enabling the determination of the maximum photochemical quantum yield of PSII (*F*_v_*/F*_m_); more recently, it was proposed to name the ratio *F*_v_*/F*_m_ as PSII activity [32]. To evaluate the efficiency of light energy utilization, continuous actinic light (190 µmol photons/m^2^/s) was applied with saturating pulses, every 20 s for 5 min. During this, the minimum and maximum fluorescence in the light (*F*′_0_ and *F*′_m_, respectively) was measured. From these values, the proportion of absorbed light used in photochemistry (ΦPSII), the electron transport rate through PSII (*ETR*), and the non-photochemical quenching (*NPQ*) were calculated using the WinControl software (version 3.2.2). All parameters were determined in ten replicates, for each condition and independent experiment.

### 2.7. Mitochondrial Membrane Potential Assessment

The probe 3,3′-dihexyloxacarbocyanine iodide [DiOC_6_(3)] (Sigma-Aldrich) was used to assess mitochondrial membrane potential (MMP) [33]. Thus, algal cells (1 × 10^6^ cells/mL) were incubated with 2.5 μmol/L DiOC_6_(3), for 10 min, at room temperature, in the dark. As a negative control, cells were treated with 50 μmol/L carbonyl cyanide 3-chlorophenylhydrazone (CCCP, Sigma-Aldrich), for 10 min, prior to DiOC_6_(3) staining, as described above. The CCCP stock solution (5 mmol/L) was prepared in DMSO, resulting in a final solvent concentration of ≤1% (*v*/*v*) in the negative control. Fluorescence intensity was quantified as described in the metabolic activity assessment (Section 2.5), in quintuplicate, for each condition and independent experiment.

### 2.8. Determination of Oxidative Stress Biomarkers

The production of reactive oxygen species (ROS) was quantified using 2′,7′–dichlorodihydrofluorescein diacetate (H_2_DCFDA, Sigma-Aldrich), as described by Machado and Soares [34]. Thus, algal cells (1 × 10^6^ cells/mL) were incubated with 10 µmol/L of H_2_DCFDA, for 90 min, in the dark, at 25 °C. Fluorescence intensity was recorded as described above (Section 2.5), in quintuplicate, for each condition and independent experiment.

Lipid peroxidation was quantified using the thiobarbituric acid reactive substance (TBARS) assay [35], following the method described by Buege and Aust [36]. Briefly, 0.5 mL of algal suspension (1 × 10^8^ cells/mL) was mixed with 1.0 mL of a reagent consisting of 15% (*w*/*v*) trichloroacetic acid (TCA), 0.375% (*w*/*v*) thiobarbituric acid (TBA), and 0.25 mmol/L HCl. The resulting mixture was heated in a bath at 95 °C, for 45 min. Following incubation, the sample was cooled to room temperature and then centrifuged at (2500× *g*, 15 min) to eliminate any potential interferences. The absorbance of the supernatant was measured at 532 nm, and the concentration of thiobarbituric acid reactive substances (TBARSs) was calculated as malondialdehyde (MDA) equivalents. MDA levels were determined using a specific extinction coefficient for the MDA-TBA adduct of 156/mM/cm [36], and results are expressed as nmol MDA per 10^6^ cells. TBARS concentrations were determined in quadruplicate for each condition and independent experiment.

### 2.9. Reproducibility and Statistical Analysis of the Results

The results are expressed as the mean ± standard deviation of three independent experiments conducted under identical conditions. Statistical difference between control and FLX-treated cells was assessed using the unpaired Student’s *t*-test; *p* values < 0.05 were considered statistically significant. Statistical treatment was performed using MS Excel (Office 365).

## 3. Results

### 3.1. Algal Growth Inhibition

**Figure 1:**
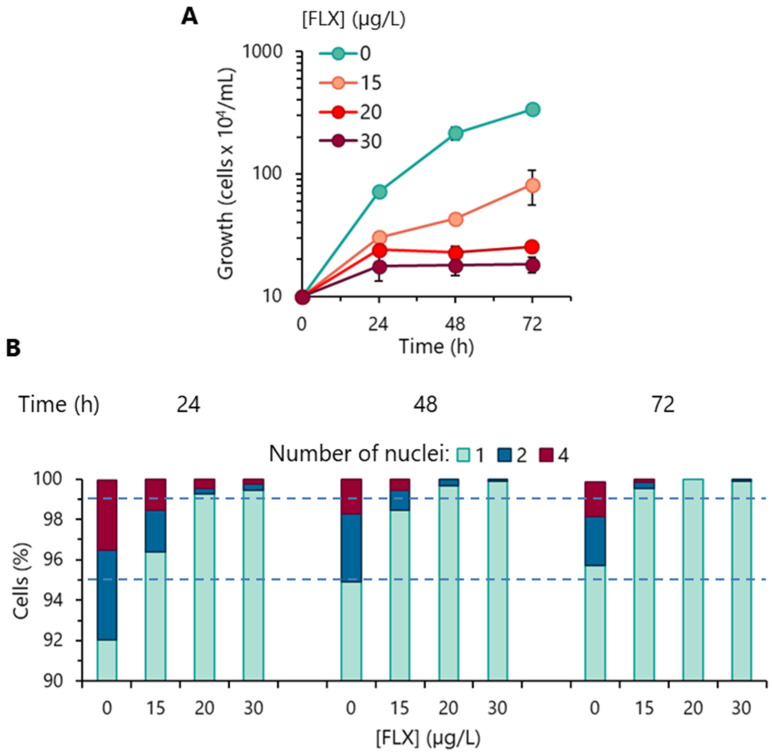
Effect of fluoxetine (FLX) on the proliferative capacity of *R. subcapitata*. (**A**) Evolution of cell concentration in OECD medium. (**B**) Analysis of the progression of the algal cell-division cycle (assessed by quantifying the number of nuclei). Data are presented as mean values of three independent experiences.

The impact of FLX on *R. subcapitata* proliferation capacity was evaluated over a period of 72 h. Algae exposed to 15 µg/L FLX grew more slowly (doubling time, td = 23 h), compared to the control (absence of FLX; td = 11 h), and showed a reduction in their population after 72 h of incubation with the pharmaceutical compound. The incubation of algal cells with 20 and 30 µg/L FLX had a deep impact on its growth; in such conditions, algal growth was practically arrested at 24 h (Figure 1A). These results prompted us to analyze the effect of FLX on different targets of the algal cell.

### 3.2. Cell-Cycle Progression Analysis

*R. subcapitata* divides asexually, through autospores. In this process, two nuclear divisions occur followed by the release of four autospores [24]. The analysis of cell-cycle progression, carried out by quantifying the number of nuclei of algal cells, showed that in the absence of the pharmaceutical (control), throughout the exponential growth (24–48 h), the population consisted of algae with 1 (mostly), 2, and 4 nuclei (Figure 1B); the entry into the stationary phase (72 h) led to enrichment of the population (>95%) with cells with a single nucleus (Figure 1B). Exposure of *R. subcapitata* to 15–30 µg/L FLX resulted in populations containing more than 95% of cells with a single nucleus earlier, i.e., at 24 h. For higher FLX concentrations (20 and 30 µg/L), the fraction of the population (>99%) consisting of a single nucleus at 24 h of exposure to FLX was remarkable (Figure 1B). These results are consistent with the growth arrest observed at 24 h in algae exposed to 20 and 30 µg/L FLX (Figure 1A).

The analysis of the results presented above strongly suggests that the presence of FLX prevented the cell-cycle progression of *R. subcapitata*, during the first nuclear division, resulting in the inhibition of algal proliferation and the production of populations composed, practically, of a single nucleus.

### 3.3. Impact on Cell Membrane Integrity

**Figure 2:**
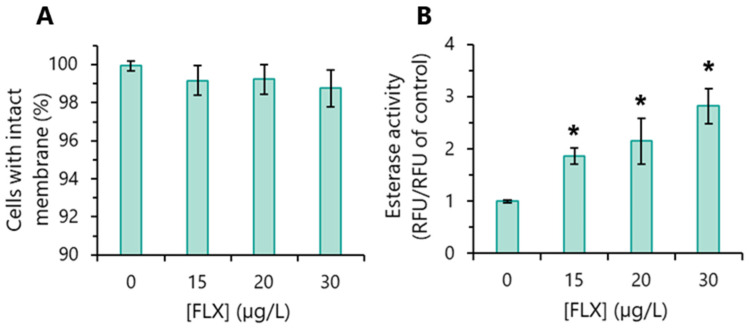
The impact of fluoxetine (FLX) on *R. subcapitata* physiology. Algae were incubated with different concentrations of FLX for 72 h in the OECD medium. (**A**) Cell membrane integrity. (**B**) Global metabolic activity of algal cells (esterase assay). Data are presented as mean values ± standard deviations (error bars). The statistical difference between control and FLX-treated cells was tested using the unpaired Student’s *t*-test; the means with (*) are significantly different from the control (*p* < 0.05).

The assessment of the effect of FLX on cell membrane permeability revealed that, up to the highest concentration tested (30 µg/L), this antidepressant had (practically) no impact. In all FLX concentrations tested, the % of cells with a disrupted membrane was ≤1% (Figure 2A). Taken together, the results obtained indicate that FLX had an algistatic effect on *R. subcapitata*: impairment of algae proliferation (growth inhibition) without cell death (assessed by the integrity of the cell membrane).

### 3.4. Overall Metabolic Activity Assessment

The ability of esterase enzymes to hydrolyse the FDA substrate has been used to measure micro-algal esterase activity and, consequently, the overall metabolic activity of *R. subcapitata* [29,37]. Thus, algae incubated with FLX showed a significantly higher esterase activity than the control. The increase in the metabolic activity was dependent on FLX concentration. For example, *R. subcapitata* cells exposed to 30 µg/L presented an increase in metabolic activity of ~3 times, compared to the control (Figure 2B).

An abiotic assay, carried out in the absence of algae, showed that FLX does not hydrolyse the FDA substrate; therefore, the increase in fluorescence intensity was due to the intensification of algal metabolic activity induced by FLX.

### 3.5. Photosynthetic Pigments Content

**Figure 3:**
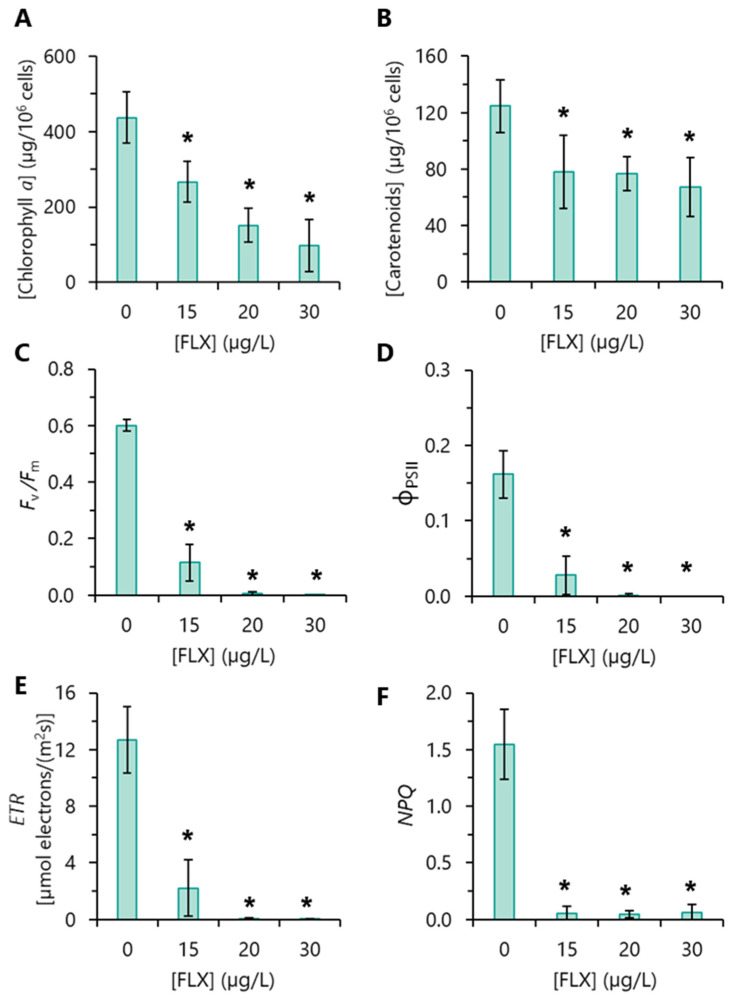
Influence of fluoxetine (FLX) on the concentration of photosynthetic pigments and photosynthetic performance of *R. subcapitata*. Algae were incubated with different concentrations of FLX for 72 h in the OECD medium. (**A**) Chlorophyl *a*. (**B**) Carotenoids. (**C**) Maximum photochemical quantum yield of PSII (*F*_v_/*F*_m_). (**D**) Effective photochemical quantum yield of PSII (ΦPSII). (**E**) Electron transport rate (*ETR*). (**F**) Non-photochemical quenching (*NPQ*). The statistical difference between control and FLX-treated cells was tested using the unpaired Student’s *t*-test; the means with (*) are significantly different from the control (*p* < 0.05).

The quantification of chlorophyll *a* (chl*a*) content revealed that FLX induced a chlorotic effect on *R. subcapitata* cells. Thus, a sharp reduction in chl*a* was observed with the increase in FLX concentration. The most severe impact was observed for 30 µg/L FLX, with an average decrease in chl*a* content of 78% (Figure 3A).

Carotenoids (car) content was also reduced in algae exposed to FLX (Figure 3B). However, the decrease was smaller than that observed with chl*a*. The reduction in car content in cells exposed to 20 or 30 µg/L FLX averaged 39% or 46%, respectively, compared with the control.

### 3.6. Photosynthetic Performance

The photosynthetic performance of algal cells exposed to FLX was analyzed by PAM. In control cultures, the maximum photochemical quantum yield of PSII (*F*_v_/*F*_m_), a parameter that gives a measure of the maximum capacity to convert light energy into chemical energy [38], presented a value close to 0.62 (Figure 3C), which is within the figures reported in the literature for *R. subcapitata* [28,39]. Algae exposed to 15 µg/L FLX presented a reduction of 81% of *F*_v_*/F*_m_, which shows that photosynthetic activity was deeply affected by FLX. At higher concentrations (20 or 30 µg/L FLX), *F*_v_/*F*_m_ was practically abolished (Figure 3C). A similar impact was observed in the other photochemical parameters: effective photochemical quantum yield of PSII (ΦPSII) (Figure 3D) and photosynthetic electron transport rate (*ETR*) (Figure 3E). The capacity of non-photochemical quenching (*NPQ*) of PSII was practically abolished for all FLX concentrations tested (Figure 3F).

### 3.7. Mitochondrial Function

The impact of FLX on the mitochondrial functionality of *R. subcapitata* was assessed through the evaluation of inner mitochondrial membrane potential (MMP), using the sensitive probe DiOC_6_(3). CCCP was used as a negative control (depolarizing agent).

**Figure 4:**
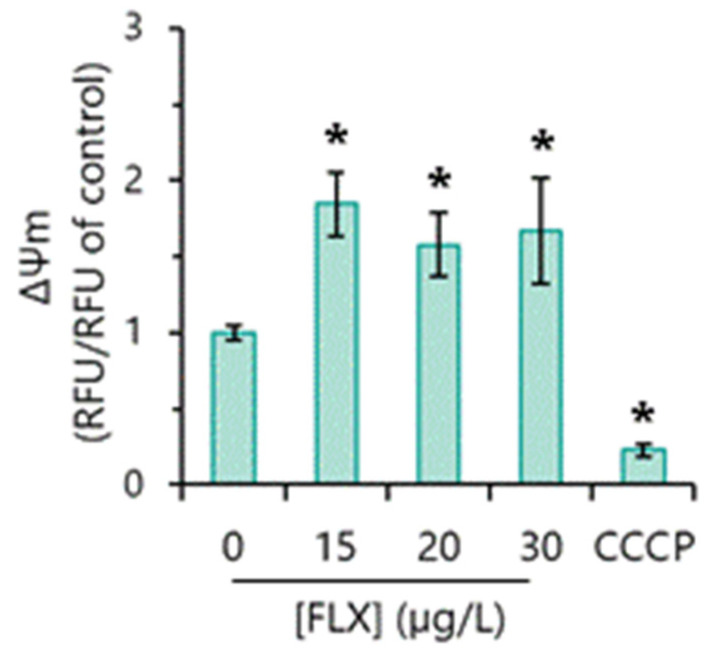
Consequences of the presence of fluoxetine (FLX) on the inner mitochondrial membrane potential (Δψm) of *R. subcapitata*. Algae were incubated with different concentrations of FLX for 72 h in the OECD medium. CCCP was used as a depolarizing agent. The statistical difference between the control and FLX or CCCP-treated cells was tested using the unpaired Student’s *t*-test; the means with (*) are significantly different from the control (*p* < 0.05).

The exposure of the algae to FLX in a concentration range between 15 and 30 µg/L induced an increase (hyperpolarization) in the MMP (Figure 4). These results suggest that FLX disturbs the mitochondrial function of the microalga *R. subcapitata*.

### 3.8. Oxidative Stress Markers Detection

**Figure 5:**
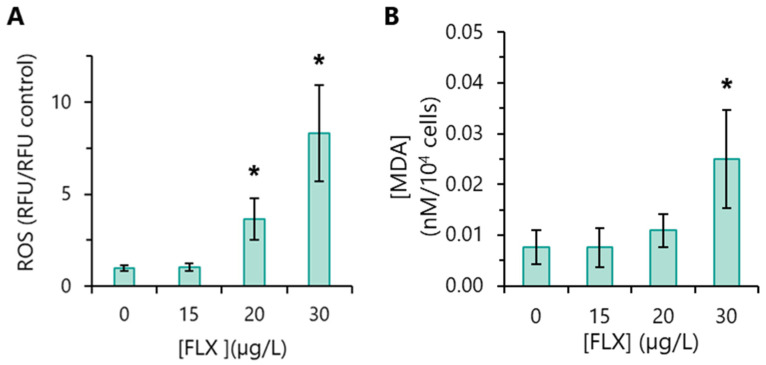
Repercussion of the presence of fluoxetine (FLX) in the appearance of oxidative stress markers in *R. subcapitata*. Algae were incubated with different concentrations of FLX for 72 h in the OECD medium. (**A**) Intracellular accumulation of reactive oxygen species (ROS). (**B**) Malondialdehyde (MDA) content (lipid peroxidation). The statistical difference between control and FLX-treated cells was tested using the unpaired Student’s *t*-test; the means with (*) are significantly different from the control (*p* < 0.05).

The assessment of intracellular accumulation of reactive oxygen species (ROS) constitutes one of the most secure markers of oxidative stress. Algae exposed to 15 µg/L FLX did not display intracellular ROS levels significantly different from the basal levels of the control. However, significantly higher levels of ROS were detected in algae incubated with 20 or 30 µg/L FLX, especially at the latter FLX concentration (Figure 5A).

One of the most common negative effects of ROS is the peroxidation of polyunsaturated fatty acids (PUFAs) present on membranes [40]. Therefore, in this study, lipid peroxidation was evaluated through the quantification of the levels of malondialdehyde, MDA (the main end-product of lipid oxidation). Algae incubated with 15 and 20 µg/L FLX did not show enhanced levels of MDA, compared to the control. However, algae exposed to 30 µg/L FLX presented an increment (~3.5 times, on average) in MDA, relative to the control (Figure 5B). These results are in agreement with those obtained in the analysis of intracellular ROS levels in the presence of 30 µg/L FLX, i.e., the presence of higher levels of ROS (Figure 5A) is consistent with the observed lipid peroxidation (Figure 5B).

## 4. Discussion

The exposure of *R. subcapitata* for 72 h to low FLX concentrations (15–30 µg/L) deeply impacted the proliferation capacity of the alga, practically inhibiting its growth at higher concentrations. Under the conditions used here the alga was slightly more sensitive to FLX, compared to other works where EC_50_ values in the range of 24–45 µg/L have been described [13,19]. It is worth highlighting the high sensitivity of *R. subcapitata* to FLX (Supplementary Material, Table S1); this alga is 10 to 100 times more sensitive to FLX than *Chlorella pyrenoidosa* [22] and *Chlorella vulgaris* [19], respectively.

Cell cycle analysis (through the quantification of the number of nuclei) revealed the accumulation of cells containing a single nucleus in algae exposed to FLX. This result is probably due to the inhibition of the first nuclear division in cells exposed to the pharmaceutical. A similar effect was observed in *R. subcapitata* cells chronologically aged [28] or exposed to Cr(VI) or Cu(II) [41]. The blockade of the first nuclear division probably led to the cessation of the cell-division cycle of the algae, which resulted in the slowdown/arrest of the growth of *R. subcapitata* incubated with FLX.

FLX induced a reduction in R*. subcapitata* pigments in a concentration-dependent manner, particularly chl*a*, the principal photosynthetic pigment of green alga [42]. The reduction in alga chl*a* content may be due to the inhibition of its biosynthesis or its degradation. In algae exposed to 15 µg/L FLX, both mechanisms can be considered. However, in the case of algae exposed to higher concentrations (20 and 30 µg/L) of FLX, considering the algal growth inhibition, the reduction in the chl*a* content in these cells should most likely be attributed to its degradation. Compatible with this possibility, an increase in the amount of pheophytin (a chl*a* degradation product) in the algae *P. tricornutum* exposed to 80 µg/L FLX has been described [23]. It was also shown that FLX brought a diminution of car pigments in *R. subcapitata*, albeit to a lesser extent than chl*a*. Similarly, a decrease in chl*a* and car content was described in the *C. pyrenoidosa*; however, this effect was only observed when this microalga was exposed to higher FLX concentrations (≥200 µg/L) [22], compared to those used in the present work, which, again, shows the greater sensitivity of *R. subcapitata* to pollutants.

It is known that chlorophylls have an essential function in light absorption as well as in energy transfer to the reaction centres of photosystems [42]. Thus, the reduction in chla content in algae incubated with FLX could have been the basis for the deterioration in PSII, which in turn could explain the deep impact of FLX on *R. subcapitata* photosynthetic performance, reflected by a decline in the maximum photochemical quantum yield (*F*_v_*/F*_m_) and the effective photosynthetic efficiency of PSII (ΦPSII) observed. A decrease in *F*_v_*/F*_m_ and ΦPSII in *C. pyrenoidosa* exposed to high (≥50 µg/L) FLX concentrations, for 4 days, was described [22]. In the same way, *P. tricornutum* exposed to 40–80 µg/L FLX, for 48 h, also showed a compromised photochemical process [23]. Photosynthetic electron transport (*ETR*) was harshly reduced in *P. subcapitata* exposed to FLX, implying less energy directed to photosynthesis.

Carotenoids have a dual role: as accessory pigments (to maximize light-harvesting) and as antioxidants, shielding the photosynthetic reaction centres by scavenging ROS or by *NPQ*, enhancing energy dissipation as heat in the last case [43]. The reduction in car content in *R. subcapitata* exposed to FLX could be linked to the observed decline in the protection of reaction centres by *NPQ*.

MMP conservation is essential for the preservation of cellular bioactivity, namely, ATP synthesis. Thus, MMP alteration is commonly seen as an indicator of mitochondrial dysfunction [44]. Here, it was observed that FLX induced a hyperpolarization of MMP, which suggests a negative impact (toxic effect) on the mitochondrial function of *R. subcapitata*. The mitochondrial perturbation, associated with the strong reduction in photosynthetic activity, could be behind an energy reduction (ATP) in the microalgae. Compatible with this possibility, it has been described that FLX causes inhibition of F1.Fo-ATPase activity and decreases the rate of ATP synthesis in mitochondria from mammalian cells [45,46,47].

*R. subcapitata* algal cells exposed to FLX seem to have their metabolism deeply disturbed, as indicated by increased esterase activity. Similarly, an increase in esterase (metabolic) activity was observed in *R. subcapitata* exposed to erythromycin [33].

FLX induced the intracellular accumulation of ROS in *R. subcapitata* at the higher concentrations tested (20 and 30 µg/L). In addition, the level of MDA (a common oxidative stress biomarker) was increased in cells exposed to 30 µg/L FLX. The levels of ROS FLX induced in *R. subcapitata* were probably not enough to cause an appreciable peroxidation of cell-membrane lipids. Consistent with this possibility, practically no loss of cell membrane integrity was observed in algae exposed up to 30 µg/L FLX. Increased levels of MDA were also observed on *P. tricornutum* and *C. pyrenoidosa* only at high FLX concentrations (≥80 µg/L or ≥200 µg/L), respectively [22,23].

**Figure 6:**
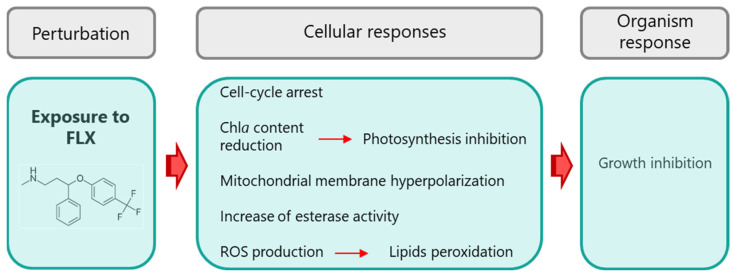
Proposal of mechanism of action (toxicity pathway) of fluoxetine on *R. subcapitata*.

Considering all the results presented, it is possible to build the main lines that can explain the toxicity mechanism of FLX in the green microalgae *R. subcapitata* (Figure 6). FLX induced the alteration of metabolism that resulted in increased esterase activity and reduction in photosynthetic pigments (mainly chl*a*). The diminution of chl*a* is probably at the origin of the deep loss of algal photosynthetic performance, explained by the decrease in *F*_v_*/F*_m_, ΦPSII, and *ETR*, while the reduction in carotenoids could be associated with the decline in protection of photosynthetic reaction centres by *NPQ*. The damage of the photosynthetic apparatus, together with the alteration of mitochondrial activity, could have led to a reduction in the energy level of the algae, which may explain the blockage of the first nuclear division, and, in the end, the arrest of algae growth. FLX also induced an intracellular accumulation of ROS and some lipid peroxidation, with a marginal (1%) loss of cell membrane integrity.

## 5. Conclusions

This study demonstrated that the antidepressant FLX exerts a toxic effect on the algae *R. subcapitata*, even at low concentrations (at the level of µg/L). The multiplicity of targets used to characterize the toxic action of FLX allowed us to propose a mechanism of toxicity in this alga. Considering the ecological relevance of *R. subcapitata*, this work offers new information about the negative impact of FLX in the aquatic environment; this data, in the future, may be useful in decision making by environmental resource managers.

## Data Availability

Data will be made available on request.

## Supplementary Materials

The following supporting information can be downloaded at https://www.mdpi.com/article/10.3390/toxics13100876/s1, Table S1: Effect concentrations (ECs) of fluoxetine (FLX) on diverse microalgae. (References [12,13,15,19,20,22,23] are cited in the Supplementary Materials).
