# Supplementary material for: The Unhappy Effects of the Antidepressant Fluoxetine on the Freshwater Microalga Raphidocelis subcapitata
Source: Toxics. 2025 Oct 14;13(10):876. doi: 10.3390/toxics13100876 (PMC12568130; doi:10.3390/toxics13100876)
Supplement: Supplementary file 1 [file toxics-13-00876-s001.zip › toxics-3885175-supplementary.pdf]

Toxics

Supplementary Material

The unhappy effects of the antidepressant fluoxetine on the freshwater microalga *Raphidocelis subcapitata*

Manuela D. Machado and Eduardo V. Soares

CONTENTS

Table S1. Effect concentrations (EC) of fluoxetine (FLX) on different microalgae S2

References S3

Table S1. Effect concentrations (EC) of fluoxetine (FLX) on diverse microalgae.

**Microalga**	**Group**	**EC_{50}** (µg/L)	**Time** (h)	**Reference**
Chlorella pyrenoidosa	Green alga	464	96	[22]
Chlorella vulgaris	Green alga	4339	96	[19]
Dunaliella tertiolecta	Green alga	170	96	[20]
Phaeodactylum tricornutum	Diatom	47	48	[23]
Raphidocelis subcapitata	Green alga	24	96	[12]
		24-39	120	[13]
		45	96	[19]
Scenedesmus acutus	Green alga	91	96	[19]
Scenedesmus quadricauda	Green alga	213	96	[19]
Skeletonema marinoi	Diatom	43	72	[15]

EC_{50} - concentration of FLX (µg/L) that induced an inhibition of 50% of algal growth

References

12. Brooks, B.W.; Foran, C.M.; Richards, S.M.; Weston, J.; Turner, P.K.; Stanley, J.K.; Solomon, K.R.; Slattery, M.; La Point, T.W. Aquatic ecotoxicology of fluoxetine. *Toxicol. Lett.* **2003**, *142*, 169–183, doi:10.1016/S0378-4274(03)00066-3.

13. Brooks, B.W.; Turner, P.K.; Stanley, J.K.; Weston, J.J.; Glidewell, E.A.; Foran, C.M.; Slattery, M.; La Point, T.W.; Huggett, D.B. Waterborne and sediment toxicity of fluoxetine to select organisms. *Chemosphere* **2003**, *52*, 135–142, doi:10.1016/S0045-6535(03)00103-6.

15. Minguez, L.; Pedelucq, J.; Farcy, E.; Ballandonne, C.; Budzinski, H.; Halm-Lemeille, M.-P. Toxicities of 48 pharmaceuticals and their freshwater and marine environmental assessment in Northwestern France. *Environ. Sci. Pollut. Res.* **2016**, *23*, 4992–5001, doi:10.1007/s11356-014-3662-5.

19. Johnson, D.J.; Sanderson, H.; Brain, R.A.; Wilson, C.J.; Solomon, K.R. Toxicity and hazard of selective serotonin reuptake inhibitor antidepressants fluoxetine, fluvoxamine, and sertraline to algae. *Ecotoxicol. Environ. Saf.* **2007**, *67*, 128–139, doi:10.1016/j.ecoenv.2006.03.016.

20. DeLorenzo, M.E.; Fleming, J. Individual and mixture effects of selected pharmaceuticals and personal care products on the marine phytoplankton species *Dunaliella tertiolecta*. *Arch. Environ. Contam. Toxicol.* **2008**, *54*, 203–210, doi:10.1007/s00244-007-9032-2.

22. Xie, Z.; Wang, X.; Gan, Y.; Cheng, H.; Fan, S.; Li, X.; Tang, J. Ecotoxicological Effects of the antidepressant fluoxetine and its removal by the typical freshwater microalgae *Chlorella pyrenoidosa*. *Ecotoxicol. Environ. Saf.* **2022**, *244*, 114045, doi:10.1016/j.ecoenv.2022.114045.

23. Feijão, E.; Cruz de Carvalho, R.; Duarte, I.A.; Matos, A.R.; Cabrita, M.T.; Novais, S.C.; Lemos, M.F.L.; Caçador, I.; Marques, J.C.; Reis-Santos, P.; et al. Fluoxetine arrests growth of the model diatom *Phaeodactylum tricornutum* by increasing oxidative stress and altering energetic and lipid metabolism. *Front. Microbiol.* **2020**, *11*, 1803, doi:10.3389/fmicb.2020.01803.
